# Feasibility and Efficacy of Intensive Dialectical Behavior Therapy Skills Training in An Outpatient Setting for A Group of Patients with Extensive Care Needs - A Transdiagnostic Approach

**DOI:** 10.1007/s11126-023-10052-9

**Published:** 2023-10-04

**Authors:** Christina Bertholds Felix, Peter Sand

**Affiliations:** 1 Department of Psychiatry for Affective Disorders, Sahlgrenska University Hospital, Gothenburg, Sweden; 2 Department of Psychology, University of Gothenburg, Gothenburg, Sweden

**Keywords:** Psychiatry, DBT skills training, Dialectical behavior therapy, CORE-OM, Psychiatric healthcare consumption, Transdiagnostic

## Abstract

**Purpose:**

Dialectical behavior therapy (DBT) is a treatment originally developed för chronically suicidal adults. It is common to adapt it by using one specific component, the DBT skills training (DBT-ST) and apply it in a group therapy setting for a variety of mental disorders. The primary aim of the study was to explore whether patients with extended care needs would report improved mental health after participating in an intensive form of DBT-ST. The secondary aim was to explore whether the use of psychiatric inpatient care for the group would decrease.

**Methods:**

Thirty-seven participants completed the Clinical Outcomes in Routine Evaluation - Outcome Measure (CORE-OM), and visual analogue scale (VAS) at three time points: pre-intervention, post-intervention and at 6-month follow-up after intensive DBT-ST.

**Results:**

One-way ANOVA showed a significant effect for time on the CORE-OM: F (2,35) = 7.93, p = .001, η^2^ = 0.312 (large effect size). Post hoc tests indicated a significant difference between pre-intervention and post-intervention (p = .001) and between pre-intervention and follow-up (p = .01). A Friedman test indicated a statistically significant difference in the VAS scale scores across the three time points, with p-values between 0.00 and 0.05. There was no difference in psychiatric healthcare consumption.

**Conclusion:**

These study results confirm to some extent the feasibility and effectiveness of the intensive DBT-ST in a transdiagnostic clinical setting. The participants had a positive outcome from the skills training program, but psychiatric healthcare consumption did not decrease.

## Introduction

### Dialectical Behavioral Therapy

Dialectical behavioral therapy (DBT) is an evidence-based treatment originally designed for suicidal women with borderline personality disorder (BPD) [1]. This disorder is characterized by dysfunctional emotion regulation, relationship difficulties, lack of impulse control, and repeated self-harm and suicidal behaviors [2]. One main purpose of DBT is to replace harmful behaviors with more effective coping strategies by teaching the patient new skills. In its standard form, DBT includes weekly individual therapy sessions in combination with skills taught in a group, weekly consultation team meetings for therapists, patient access to a therapist between sessions, and involvement of the patient’s family and network. The essence of DBT is finding a balance between acceptance and change-based strategies. The acceptance strategies are based on the principles of Zen Buddhism and the change strategies on the principle of behaviorism, with cognitive behavior therapy interventions such as exposure, problem-solving, and contingency management [1]. DBT has been developed and adapted to several psychiatric conditions in addition to BPD, all with emotional regulation difficulties as a common factor [3, 4]. Studies show that DBT has a positive effect on anger and aggressive behavior [5], eating disorders [6], substance use [7], bipolar disorder [8], depression in the presence of personality disorder [9], and post-traumatic stress disorder [10]. Linehan’s biosocial model is a primary theory in DBT which emphasizes the underlying factor in BPD as emotional dysregulation [1]. In recent years, an increasing number of studies have focused on emotional dysregulation as a common factor across a range of psychological disorders and studies have shown it to be a successful way to help psychiatric patients [11–14]. Linehan emphasized that hospitalization for patients with BPD should be as brief as possible and that patients with BPD are unlikely to recover as a result of hospital admission. There is also a high risk of recurring admissions för this patient group. Therefore it is important to reach this group via outpatient interventions, which DBT does [1, 15].

### DBT Skills Training (DBT-ST)

Standard DBT treatment is costly and clinical availability is limited; interventions where the DBT skills training component (DBT-ST) alone is offered have therefore become increasingly common [16]. DBT-ST is given in groups and involves four skills training modules: emotional regulation, distress tolerance, interpersonal effectiveness, and mindfulness [1]. A systematic review by Valentine et al. [16] examined 17 studies where DBT-ST was offered as a stand-alone treatment. The results showed preliminary evidence that DBT-ST was promising for a broad patient population, but methodological shortcomings made it difficult to draw definite conclusions. Valentine et al. [16] emphasized that post hoc analyses of randomized clinical trial data from major DBT studies have found that the skills training is a significant treatment component for reducing mental illness. Further examples of conditions where DBT-ST has been shown to have a positive effect are depression [4, 17, 18], suicide attempts and intentions [4, 19, 20], non-suicidal self-injury (NSSI) episodes [4], overweight [21], anxiety disorder and emotion dysregulation problems [4, 18, 22], bipolar disorder type 1 [23], substance dependence [24–26], and ADHD symptoms [27, 28]. The DBT-ST treatment programs are often adjusted for specific patient groups, for example adding psychoeducation and selecting modules that should be prioritized [16, 26].

Although many DBT-ST treatments are aimed at specific patient groups (e.g. ADHD, depression, substance abuse) [16, 26], in the present study, we have instead chosen to target a broad patient group with few exclusion criteria, a pragmatic approach that allows the DBT-ST unit to serve several different units at the clinic. The target group is patients whom psychiatric outpatient clinics have not been able to treat through their usual range of treatment and/or who are in the risk zone for long or multiple admissions to inpatient psychiatric care. We have also chosen to offer DBT-ST with a new intensity. The most common intensity is about 2–3 h a week over a period of 3.5 to 6 months, totaling 17.5 to 47 h of DBT-ST [16]. Since we are targeting a patient group that has high care needs, with a low level of functioning and not infrequently previous treatment attempts that have failed, the present study offers treatment of 110 h of DBT-ST over 10 weeks. The number of hours is then close to the standard DBT arrangement, which is 130 h spread out over a year. The idea is that the much higher intensity in this study will be able to quickly break the patient’s problem behaviors and increase the chance that patients who undergo the treatment will at least acquire some basic DBT skills that can help them in future interventions. The study examines whether this arrangement is feasible in a Nordic clinical context and whether the patients actually remain in the treatment when it is offered in this compressed and intensive form.

The present study examines whether benefits of DBT-ST can be observed among diagnostically diverse participants receiving intensive DBT-ST; it also measures whether healthcare consumption decreases after intensive DBT-ST, together with self-assessment scales of well-being, psychiatric symptoms, life functioning, risk behaviors, social functioning and emotional functioning.

## Method

### Design and Statistical Analysis

The study had a within-group design and was carried out in an outpatient clinical setting, with pre-intervention measurements at baseline and post-intervention measurements at the completion of the intervention and at the 6-month follow-up. Data were analyzed primarily with ANOVA, and non-parametric tests (Friedman’s test and Wilcoxon signed-rank test) were used when the data were not normally distributed. Guidelines for interpreting the partial eta-squared are as follows: η^2^ = 0.01 indicates a small effect, η^2^ = 0.06 indicates a medium effect and η^2^ = 0.14 indicates a large effect. For interpreting Cohen’s d, the following common guidelines, proposed by Cohen [29], are used: 0.01 = small effect, 0.06 = moderate effect and 0.14 = large effect. All analyses were performed using IBM SPSS Statistics, version 28. P-values at or below 0.05 were considered significant.

### Participants

The participants were recruited at four psychiatric community outpatient clinics belonging to the department of psychiatry at Sahlgrenska University Hospital, Gothenburg, Sweden. The inclusion criteria were patients with long-term mental health problems, affect lability, high consumption (or risk of high consumption) of inpatient psychiatric care and who had previously had interventions where they dropped out or did not benefit from the treatment. The exclusion criteria were substance use disorder and acute suicidal behavior. The decision to send a referral to the unit was made by the attending psychiatrist responsible for the patient and the therapist at the respective outpatient unit.

**Fig. 1:**
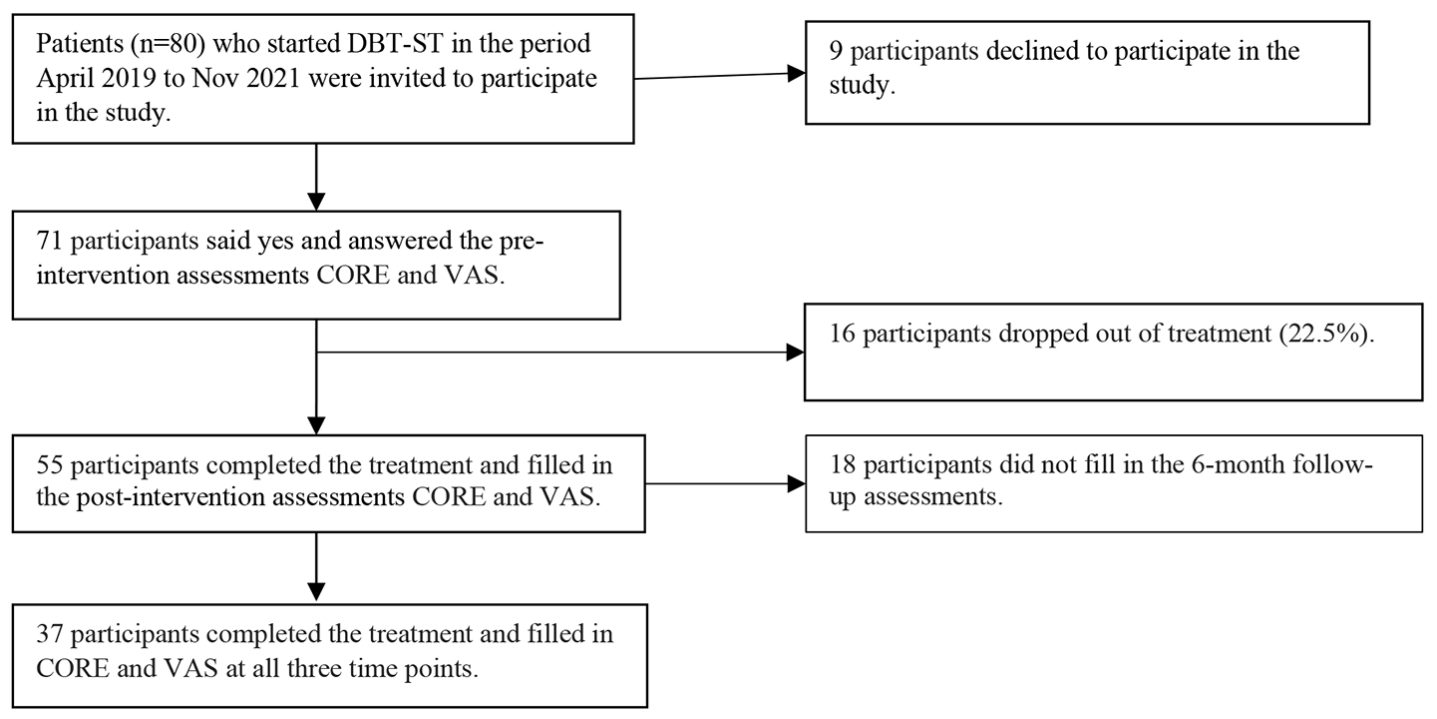
Participant flowchart

In the period April 2019 to Nov 2021, patients (n = 80) who met the inclusion criteria and had an approved referral were asked if they were interested in participating in the study. Of the 80 patients, 71 agreed and started DBT-ST and 55 completed the training. See Fig. 1 for the participant flowchart and Table 1 for the participants’ demographic characteristics.

**Table 1:** Demographic characteristics of the participants

	Entire group (n = 71)	Completed treatment ^a^ and 6-month follow-up (n = 37)	Completed treatment ^b^ (n = 55)	Dropouts (n = 16)
**Age** in years, mean (SD)	32.8 (10.8)	32.9 (11.4)	33.9 (10.8)	28.9 (10.5)
**Gender**				
Female	59 (81.9%)	29 (78.4%)	44 (80%)	14 (87.5%)
Male	13 (18.1%)	8 (21.6%)	11 (20%)	2 (12.5%)
**Education**				
Elementary	18 (25%)	13 (35.1%)	14 (25.5%)	4 (25%)
High school	38 (54%)	16 (43.2%)	29 (52.7%)	9 (56.3%)
Graduate	15 (21%)	8 (21.6%)	12 (21.8%)	3 (18.8%)
**Main diagnosis**				
Borderline personality disorder	37 (52%)	17 (45.9%)	30 (54.5%)	7 (43.8%)
Unspecified personality disorder	8 (11%)	6 (16.2%)	6 (10.9%)	2 (12.5%)
Axis 1 ^**c**^	26 (37%)	14 (37.8%)	19 (34.5%)	7 (43.8%)
**More than one diagnosis**	47 (66%)	25 (67.6%)	37 (67.3%)	11 (68.8%)
**Sick leave** (25% or more)	70 (99%)	37 (100%)	55 (100%)	16 (100%)
**Days in treatment**, of total 40 days, mean (SD)	29.5 (10.7)	32.1 (6.5)	34.1 (5.2)	13.6 (9.6)
**Number of individual sessions**, mean (SD)	7.5 (2.6)	8.0 (2.0)	8.4 (1.9)	4.6 (2.4)

^a^ *Participants who attended at least 20 out of 40 days were counted as completers.*

^b^ *Completed treatment but not the 6-month follow-up measure with CORE and VAS.*

^**c**^ *Mixed anxiety and depressive disorder, depression, GAD, OCD, eating disorder, bipolar disorder, ADHD, autism.*

### Procedure and Treatment Intervention

The treatment procedure began with a commitment phase consisting of two or three sessions with a focus on giving the patient information about the treatment, removing obstacles to participation, and formulating goals. Intensive DBT-ST took place in groups of up to 10 participants and lasted for 10 weeks, 4 days per week. The total number of skills training hours per week were 11 h. On two of the days, the training took place both in the morning and afternoon. The two other days it was only in the morning. The participants also received 30–60 min of individual consultation meetings per week. The focus of these sessions was to practice and generalize the skills according to the participant’s goals. The participants received a manual in book form describing the four skills training modules and they were given homework assignments at each group meeting, to be shared with the group at a later session. There were two skills trainers leading the group. These were closed groups, meaning that no new patients were accepted until the next group began.

The content of DBT-ST followed the standard DBT model and consisted of the following modules: *Emotion regulation* with a focus on explaining the function of emotions, reducing emotional vulnerability, emphasizing the importance of self-care, and providing skills such as problem-solving, strategies for cognitive flexibility, and “opposite action”; *Mindfulness* with a focus on paying attention to thoughts, feelings, and the environment in the present without judging and without acting impulsively; *Interpersonal effectiveness* with a focus on teaching skills to create and maintain good relationships and finding balance in relationships; lastly, *Distress tolerance* with a focus on giving the patients skills to survive crises without engaging in dangerous behaviors [1]. Table 2 shows the treatment schedule in relation to the different modules during the 10-week intervention. As is common in DBT, smaller individual adjustments were made to adapt the content to the patient’s needs, for example by bringing forward parts of the distress tolerance module to the first week. The participants filled in a self-assessment on three occasions (pre-treatment, post-treatment, and at a 6-month follow-up). They were encouraged to fill in the forms electronically, but paper sheets were also offered for those who did not have electronic means.

**Table 2:** Content of skills training, week by week

W1 Mindfulness introduction and biosocial theory	W6 Emotion regulation and interpersonal effectiveness skills
W2 Mindfulness and validation	W7 Interpersonal effectiveness
W3 Mindfulness and emotion regulation	W8 Distress tolerance
W4 Emotion regulation	W9 Distress tolerance
W5 Emotion regulation	W10 Summary

### The Skills Trainers

The outpatient unit that offered the DBT-ST was newly established and was aimed to serve four different outpatient units belonging to the psychiatric clinic. The staff recruited to carry out the intervention had the following professional background: psychologist (2), nurse (1), auxiliary psychiatric nurse (2), physiotherapist (2), occupational therapist (1), and adult educator (1). Together they completed a 10-day training course in DBT-ST and then they received external DBT guidance on a regular basis. One of the clinicians had previously worked with DBT. One-to-one consultation meetings between a participant and a skills trainer took place every week to increase adherence to the model. If staff were sick or off duty, other skills trainers covered to ensure all patients got their individual sessions. Due to the ongoing Covid-19 pandemic, certain adjustments were made for both the professional staff and the participating patients (digital attendance for those who had cold symptoms, use of face masks and appropriate distance between those attending in person).

The skills trainers took turns leading the group according to a schedule where everyone was represented, which meant that the participants did not have one and the same trainer for their group throughout the intervention. During the individual sessions, the participants met with the same skills trainer each time. Distribution of professions was then as follows (n = 71): 21% met a psychologist, 10% met a nurse, 25% met an auxiliary psychiatric nurse, 24% a physiotherapist, 11% an occupational therapist and 9% met an educator.

### Outcome Measures

#### Clinical Outcomes in Routine Evaluation - Outcome Measure, CORE-OM

The CORE-OM is a self-assessment scale consisting of 34 items that address domains of *subjective well-being* (4 items), *symptoms* (12 items: anxiety, 4 items; depression, 4 items; physical problems, 2 items; trauma, 2 items), *functioning* (12 items: general functioning, 4 items; close relationships, 4 items; social relationships, 4 items), and *risk/harm* (6 items: risk to self, 4 items; risk to others, 2 items). Items are scored on a five-point scale from 0 (not at all) to 4 (all the time). The scores for all answers are summarized, and a higher score indicates a worse mental state. The instrument is reliable and has high validity and good sensitivity to measure change. CORE-OM correlates well with several well-known assessment scales, it shows good ability to distinguish between clinical and non-clinical populations, and test–retest reliability is between 0.75 and 0.95 [30–33]. CORE-OM has been validated for Swedish conditions, with the conclusion that the psychometric properties of the Swedish version are well in line with the original [34]. Mullin et al. [35] have calculated the reliable change index (RCI) for CORE-OM and found it to be 5 points, which means that patients who score 5 points lower in post-treatment measurement than in pre-treatment measurement can be considered clinically improved by the treatment. The CORE-OM cut-off for the clinical population is 10 points [36]. Clients who meet RCI 5 and no longer belong to the clinical population are considered recovered [35].

### Self-assessment of Social and Emotional Functioning on a 100-Point Visual Analog Scale (VAS)

The patients were asked to answer how well they felt they were able to handle their mental state (depression/anxiety), their emotions, and their relationships during the last 7 days. They are instructed to put a cross on the straight line at the point that most accurately expresses their degree of agreement, where the endpoints were 0 = No ability to manage at all and 100 = Complete ability to manage. The questions were as follows: (1) I have been able to handle my mental state, (2) I have been able to handle my feelings, (3) I have been able to handle my relationships.

### Measuring Healthcare Consumption

The frequency of psychiatric healthcare consumption (6 months before the start of treatment and 6 months after the end of treatment) is measured based on the patient’s chart review, taking into account the following parameters: *Number of visits to the psychiatric emergency department*, *Number of admissions to a psychiatric hospital, Number of days in psychiatric inpatient care*.

## Results

### Treatment Outcome: CORE-OM

A one-way repeated measures ANOVA was conducted to compare scores on the CORE-OM at Time 1 (pre-intervention), Time 2 (immediately following the intervention), and Time 3 (the 6-month follow-up). There was a significant effect for time in the CORE-OM total score F (2,35) = 7.93, p = .001, η^2^ = 0.312 (large effect size). Post hoc tests indicated a significant difference both immediately after the intervention and at the 6-month follow-up, see Table 3 for more information.

There was also a significant effect for time in the CORE-OM subscales: *Subjective well-being*: F (2,35) = 6.31, p = .005, η^2^ = 0.265 (large effect size); *Life functioning*: F (2,35) = 12.81, p < .001, η^2^ = 0.423 (large effect size); *Problems/symptoms*: F (2,35) = 9.86, p < .0.001, η^2^ = 0.360 (large effect size); *Risk/harm*: F (2,35) = 3.95, p = .028, η^2^ = 0.184 (large effect size). Post hoc tests (with the Bonferroni correction for multiple tests) indicated a significant difference both immediately and at the 6-month follow-up for the subscales *Subjective well-being* and *Life functioning*. *Risk/harm* and *Problems/symptoms* showed a significant positive effect between pre- and post-intervention. The subscales *Problems/symptoms* and *Life functioning* indicated a significant negative effect *between* Time 2 and Time 3, see Table 3 for more information.

**Table 3:** Means, standard deviations, and comparisons for CORE-OM across three time points

	Time 1 *pre-intervention*	Time 2 *post-intervention*	Time 3 *follow-up*	Time 1 to time 2 (n = 55)	Time 1 to Time 3 (n = 37)	Time 2 to Time 3 (n = 37)
CORE-OM	M (SD)	M (SD)	M (SD)	Sig.^a^	Sig.^a^	Sig.^a^
**Total scale**	22.21 (5.32)	18.39 (5.91)	19.17 (5.48)	p = .001	p = .01	p = .85
**Subscales**:						
*Subjective well-being*	26.70 (6.59)	22.70 (6.05)	23.45 (7.15)	p < .001	p = .05	p = .94
*Problems/* *Symptoms*	27.73 (6.14)	23.27 (7.36)	26.53 (6.70)	p < .001	p = .92	p = .004 ^b^
*Life functioning*	20.72 (5.73)	16.06 (5.29)	18.09 (5.94)	p < .001	p = .05	p = .007 ^b^
*Risk/Harm*	11.22 (7.35)	6.37 (10.26)	8.83 (7.01)	p = .04	p = .23	p = .40

^**a**^ *Significance of the mean difference, using the Bonferroni adjustment for multiple comparisons. A p-value of 0.05 or less was considered significant.*

^b^ *Indicates a significant worsening between these two time points.*

### Treatment Outcome: VAS

The means and standard deviations of the VAS are presented in Table 4. The results of the Friedman test indicated that there was a statistically significant difference from pre-intervention to 6-month follow-up in the participants’ self-reported ability to handle their mental state, emotions and relationships (*Handle mental state*: chi-squared (2, n = 37) = 12.09, p = .002; *Handle emotions*: chi-squared (2, n = 37) = 17.79, p < .001; *Handle Relationships*: chi-squared (2, n = 37) = 11.12, p = .004). Post hoc tests (with the Bonferroni correction for multiple tests) indicated significant differences for all three variables between pre- and post-intervention and between pre-intervention and 6-month follow-up, see Table 4 for more information.

**Table 4:** Means, standard deviations, and comparisons for VAS across three time points

	Time 1 *pre-intervention*	Time 2 *post-intervention*	Time 3 *follow-up*	Time 1 to time 2 (n = 55)	Time 1 to Time 3 (n = 37)	Time 2 to Time 3 (n = 37)
**VAS**	M (SD)	M (SD)	M (SD)	Sig.^a^	Sig.^a^	Sig.^a^
Mental state	39.4 (24.1)	57.2 (24.1)	49.7 (24.8)	p < .001 Cohen’s d = 0.40	p = .05 Cohen’s d = 0.23	p = .22
Emotions	33.4 (20.4)	58.8 (24.1)	50.5 (26.3)	p < .001 Cohen’s d = 0.45	p < .001 Cohen’s d = 0.39	p = .36
Relations	44.1 (25.9)	61.4 (25.1)	55.2 (26.7)	p < .002 Cohen’s d = 0.36	p = .036 Cohen’s d = 0.24	p = .40

^**a**^ *Significance of the mean difference, using the Bonferroni adjustment for multiple comparisons. A p-value of 0.05 or less was considered significant.*

### Psychiatric Healthcare Consumption

The means and standard deviations of healthcare consumption for the patients who completed the treatment and the patients who dropped out of treatment are presented in Table 5. Wilcoxon signed-rank tests revealed no statistically significant reduction in frequency of psychiatric hospital admissions, days of psychiatric hospitalizations, or frequency of psychiatric emergency visits.

**Table 5:** Means and standard deviations for healthcare consumption 6 months pre DBT-ST and 6 months post DBT-ST for the patients who completed the treatment (n = 55) and for the patients who dropped out of treatment (n = 16)

Completers (n = 55)				Drop-outs (n = 16)		
	Number of persons ^a^	Pre DBT M (SD)	Post DBT M (SD)	Number of persons ^a^	Pre DBT M (SD)	Post DBT M (SD)
Psychiatric emergency visits	23	2.2 (2.6)	1.5 (2.4)	8	1.0 (1.1)	1.6 (1.8)
Psychiatric hospital admissions	20	1.5 (1.7)	1.3 (2.1)	5	0.6 (0.5)	0.6 (0.5)
Days of psychiatric hospitalization	20	16.2 (22.9)	7.4 (13.5)	5	4.1 (6.4)	4.8 (5.5)

^**a**^ *Number of persons using psychiatric hospital care*

## Discussion

DBT-ST is used as a standalone treatment in a variety of clinical settings and populations. However, there has been relatively little robust empirical support for using DBT-ST without the other DBT treatment components [16, 26, 37]. The primary aim of this study was to investigate whether participants with a variety of common psychiatric diagnoses in a specialized psychiatric outpatient setting would improve after 10 weeks of intensive DBT-ST, 11 h per week distributed over four days. The intensive DBT-ST was primarily intended for patients with extensive care needs, in other words, those who for various reasons were not sufficiently helped by the traditional outpatient treatment.

According to our first hypothesis, that the participants would report an improvement in their mental health, the pre–post intervention self-reported data revealed that those who completed the treatment had experienced a significant reduction in symptoms, as measured by CORE-OM and VAS. The effects were stable at the 6-month follow-up. The self-reported improvement of psychiatric symptoms was in line with other studies in the area [4, 16, 19]. Our second hypothesis, that health care consumption (number of psychiatric emergency department visits, number of psychiatric hospital admissions, and days of psychiatric hospitalization) would decrease after 10 weeks of intensive DBT-ST, revealed no significant differences pre–post intervention when comparing the period 6 months before and 6 months after the intervention. Previous research has shown reduced healthcare consumption after patients received DBT-ST; for example, such findings are found in McMain et al. [20] and Linehan et al. [4].

For the future we want further research on how to optimize and adjust the DBT-ST treatment in the best way and find the right level of treatment intensity. It would be desirable to examine the following three aspects more closely.

First, intensive DBT-ST (110 h) requires significantly greater financial resources than the less intensive variants. Considering the costs, we recommend conducting a randomized clinical trial, to compare intensive DBT-ST (110 h) with other DBT-ST protocols involving different lengths of time, intensity and frequency. The literature review by Valentine et al. [16] found that the total number of hours of DBT-ST across 17 DBT-ST studies varied dramatically (from 17.5 to 47 h). They compared it to standard DBT, which provides about 130 h of skills training over the course of a year. The variations today are many, but we know little about what is most effective. Second, we want more research to be carried out on effective components of DBT-ST. There are probably parts of DBT-ST that can be left out and other parts that are more crucial aspects of skills training. For example, Krantz et al. [38] found that the component “accept without judgment” in mindfulness was of particular importance in reducing NSSI. Valentine et al. [16] highlighted that the implementation of DBT-ST differed markedly from the clinical trials using standard DBT. They found that 59% of the 17 studies of DBT-ST offered all four modules, while 41% omitted at least one. A couple of studies also made their own additions to the treatment. Later studies confirmed these findings [21, 23]. The effects of removing and modifying parts of the skills training should be examined more closely. Finally, there are other effective evidence-based interventions today that address the role of emotion regulation for transdiagnostic patient groups, and these work with CBT interventions in a similar way to DBT-ST; unified protocol administered in groups is one such example [11]. A study with a randomized comparison between DBT-ST and group-administered unified protocol would underpin more informed decisions in the choice of intervention and would make it easier to optimize treatment.

### Strengths and Limitations

This study has some limitations. First, the lack of a control group, which is unfortunately the case with many other studies on DBT-ST [16]. A second limitation was the Covid-19 pandemic and its effects on society and health care. The clinicians had to become more flexible with the criterion of attendance, and a variety of adjustments had to be made. In particular, clients with cold symptoms were not allowed to attend in person; instead, video meetings were used. Around 20% of patients’ physical visits were replaced by online video calls while the pandemic was ongoing. The pandemic also led to increased levels of sick leave among the clinicians, who partly had to handle the skills training without a co-therapist. Finally, another limitation is that we used few self-report instruments; although the CORE-OM has been shown to be robust in measuring change [30–34], it would be useful to look more specifically at factors such as NSSI, emotion regulation ability, and suicidality. Future research would benefit from adding more precise measurements.

A strength of this study was the low dropout rate (22.5%) in comparison to other studies of DBT-ST, where the dropout rate was sometimes as high as 44–66% [18, 21, 22, 39]. Furthermore, it is interesting to compare our dropout rate with a meta-analysis of standard DBT treatments [40], where the dropout rate was 27.3% from pre-treatment to post-treatment. The present study cannot answer why the patients’ attendance was high. A concern when planning intensive DBT-ST was that the intensity would make the patients less interested in participating, but this did not happen; instead, this intensity proved feasible. Furthermore, the attendance of participants who completed the treatment (n = 55) was high: they came to the treatment on average 34.1 (SD 5.2) of 40 days. Another strength of the present study was the inclusion of a broad transdiagnostic group of patients with complex psychiatric problems, which is common in a clinical context. Finally, it is a strength that the present study reports on the staff members’ professional background and the extent of DBT training they had received. In the review of DBT-ST conducted by Valentine et al. [16], they found that clinical training in DBT varied widely across studies (from graduate coursework to a 10-day workshop), they also found that several studies provided no information on the clinicians’ training at all. These limitations reduce generalizability.

### Clinical Implications

The literature shows that the skills training component from standard DBT, DBT-ST, is used across a range of clinical settings and populations. In this study we found evidence supporting the use of intensive DBT-ST in a transdiagnostic outpatient setting for patients with complex psychiatric problems.

The study showed that intensive DBT-ST can be administered by psychiatric clinicians without previous experience of DBT as a method, provided that they receive internal training in combination with regular tutorial support and supervision.

The findings suggest that a more careful pre-assessment should be made by experienced clinicians before the 10-week training starts, to select the patients who are most in need of intensive DBT-ST in its current form of 110 h in a period of 10 weeks. It is likely that several of the participants in our study could have had the same outcome from fewer hours of skills training, especially those who initially had lower scores on CORE-OM, less healthcare consumption, and no severe personality disorder.

An important component of standard DBT is generalizing DBT skills taught in the clinic by encouraging the clients to practice these skills independently in their own natural environment. A disadvantage of the 4 days per week format is that it gives little time between the sessions to practice the skills and do the homework.

## Conclusions

Overall, it is important to consider pros and cons before offering the intensive form of DBT-ST.

An important aspect of DBT treatment is practicing DBT skills in the natural environment (outside the clinical setting); intensive DBT-ST gives less time for that in comparison with less intensive DBT-ST programs. To summarize, we can to some extent conclude that the results support the feasibility and efficacy of the intensive DBT-ST in a transdiagnostic clinical setting. Despite setbacks such as the Covid-19 pandemic, requiring minor organizational change and digital adaptations, many patients chose to complete the treatment and had a positive outcome from the skills training program. Even though we found positive results, taking the present study and previous ones into consideration, it is unclear whether the intensive form added much extra value for the participants. The study was also unable to show any reduction in the participants’ consumption of inpatient care or number of emergency visits, which we hypothesized as a possible outcome of this more intensive intervention.
